# Supplementary material for: Mapping the evolution of fertility support policies in China: A content and instrumental analysis
Source: PLoS One. 2025 Oct 9;20(10):e0332137. doi: 10.1371/journal.pone.0332137 (PMC12510515; doi:10.1371/journal.pone.0332137)
Supplement: S1 Appendix — (ZIP) [file pone.0332137.s001.zip › S1 Appendix. 226 original policy documents/169-中华人民共和国社会保险法(2018修正).doc]

中华人民共和国社会保险法(2018修正)

**发布部门：** [全国人大常委会](https://www.pkulaw.com/law/chl?Aggs.IssueDepartment=201)

**发文字号：**中华人民共和国主席令第25号

**发布日期：**2018.12.29

**实施日期：**2018.12.29

**时效性：** [现行有效](https://www.pkulaw.com/law/chl?Aggs.TimelinessDic=01)

**效力级别：** [法律](https://www.pkulaw.com/law/chl?Aggs.EffectivenessDic=XA0101)

**法规类别：** [社会福利与社会保障](https://www.pkulaw.com/law/chl?Aggs.Category=01505) [营商环境优化](https://dobusiness.pkulaw.com/)

中华人民共和国社会保险法
（2010年10月28日第十一届全国人民代表大会常务委员会第十七次会议通过　根据2018年12月29日第十三届全国人民代表大会常务委员会第七次会议《[关于修改〈中华人民共和国社会保险法〉的决定](https://www.pkulaw.com/chl/aac57b5ebad804f6bdfb.html#class=alink)》修正）

目　　录

　　第一章　总　　则
　　第二章　基本养老保险
　　第三章　基本医疗保险
　　第四章　工伤保险
　　第五章　失业保险
　　第六章　生育保险
　　第七章　社会保险费征缴
　　第八章　社会保险基金
　　第九章　社会保险经办
　　第十章　社会保险监督
　　第十一章　法律责任
　　第十二章　附　　则

第一章　总　　则

　　第一条　为了规范社会保险关系，维护公民参加社会保险和享受社会保险待遇的合法权益，使公民共享发展成果，促进社会和谐稳定，根据[宪法](https://www.pkulaw.com/chl/520e1c95bc3e633bbdfb.html#class=alink)，制定本法。

　　第二条　国家建立基本养老保险、基本医疗保险、工伤保险、失业保险、生育保险等社会保险制度，保障公民在年老、疾病、工伤、失业、生育等情况下依法从国家和社会获得物质帮助的权利。

　　第三条　社会保险制度坚持广覆盖、保基本、多层次、可持续的方针，社会保险水平应当与经济社会发展水平相适应。

　　第四条　中华人民共和国境内的用人单位和个人依法缴纳社会保险费，有权查询缴费记录、个人权益记录，要求社会保险经办机构提供社会保险咨询等相关服务。

　　个人依法享受社会保险待遇，有权监督本单位为其缴费情况。

　　第五条　县级以上人民政府将社会保险事业纳入国民经济和社会发展规划。

　　国家多渠道筹集社会保险资金。县级以上人民政府对社会保险事业给予必要的经费支持。

　　国家通过税收优惠政策支持社会保险事业。

　　第六条　国家对社会保险基金实行严格监管。

　　国务院和省、自治区、直辖市人民政府建立健全社会保险基金监督管理制度，保障社会保险基金安全、有效运行。

　　县级以上人民政府采取措施，鼓励和支持社会各方面参与社会保险基金的监督。

　　第七条　国务院社会保险行政部门负责全国的社会保险管理工作，国务院其他有关部门在各自的职责范围内负责有关的社会保险工作。

　　县级以上地方人民政府社会保险行政部门负责本行政区域的社会保险管理工作，县级以上地方人民政府其他有关部门在各自的职责范围内负责有关的社会保险工作。

　　第八条　社会保险经办机构提供社会保险服务，负责社会保险登记、个人权益记录、社会保险待遇支付等工作。

　　第九条　工会依法维护职工的合法权益，有权参与社会保险重大事项的研究，参加社会保险监督委员会，对与职工社会保险权益有关的事项进行监督。

第二章　基本养老保险

　　第十条　职工应当参加基本养老保险，由用人单位和职工共同缴纳基本养老保险费。

　　无雇工的个体工商户、未在用人单位参加基本养老保险的非全日制从业人员以及其他灵活就业人员可以参加基本养老保险，由个人缴纳基本养老保险费。

　　公务员和参照[公务员法](https://www.pkulaw.com/chl/7f4dfcf8c72758a8bdfb.html#class=alink)管理的工作人员养老保险的办法由国务院规定。

　　第十一条　基本养老保险实行社会统筹与个人账户相结合。

　　基本养老保险基金由用人单位和个人缴费以及政府补贴等组成。

　　第十二条　用人单位应当按照国家规定的本单位职工工资总额的比例缴纳基本养老保险费，记入基本养老保险统筹基金。

　　职工应当按照国家规定的本人工资的比例缴纳基本养老保险费，记入个人账户。

　　无雇工的个体工商户、未在用人单位参加基本养老保险的非全日制从业人员以及其他灵活就业人员参加基本养老保险的，应当按照国家规定缴纳基本养老保险费，分别记入基本养老保险统筹基金和个人账户。

　　第十三条　国有企业、事业单位职工参加基本养老保险前，视同缴费年限期间应当缴纳的基本养老保险费由政府承担。

　　基本养老保险基金出现支付不足时，政府给予补贴。

　　第十四条　个人账户不得提前支取，记账利率不得低于银行定期存款利率，免征利息税。个人死亡的，个人账户余额可以继承。

　　第十五条　基本养老金由统筹养老金和个人账户养老金组成。

　　基本养老金根据个人累计缴费年限、缴费工资、当地职工平均工资、个人账户金额、城镇人口平均预期寿命等因素确定。

　　第十六条　参加基本养老保险的个人，达到法定退休年龄时累计缴费满十五年的，按月领取基本养老金。

　　参加基本养老保险的个人，达到法定退休年龄时累计缴费不足十五年的，可以缴费至满十五年，按月领取基本养老金；也可以转入新型农村社会养老保险或者城镇居民社会养老保险，按照国务院规定享受相应的养老保险待遇。

　　第十七条　参加基本养老保险的个人，因病或者非因工死亡的，其遗属可以领取丧葬补助金和抚恤金；在未达到法定退休年龄时因病或者非因工致残完全丧失劳动能力的，可以领取病残津贴。所需资金从基本养老保险基金中支付。

　　第十八条　国家建立基本养老金正常调整机制。根据职工平均工资增长、物价上涨情况，适时提高基本养老保险待遇水平。

　　第十九条　个人跨统筹地区就业的，其基本养老保险关系随本人转移，缴费年限累计计算。个人达到法定退休年龄时，基本养老金分段计算、统一支付。具体办法由国务院规定。

　　第二十条　国家建立和完善新型农村社会养老保险制度。

　　新型农村社会养老保险实行个人缴费、集体补助和政府补贴相结合。

　　第二十一条　新型农村社会养老保险待遇由基础养老金和个人账户养老金组成。

　　参加新型农村社会养老保险的农村居民，符合国家规定条件的，按月领取新型农村社会养老保险待遇。

　　第二十二条　国家建立和完善城镇居民社会养老保险制度。

　　省、自治区、直辖市人民政府根据实际情况，可以将城镇居民社会养老保险和新型农村社会养老保险合并实施。

第三章　基本医疗保险

　　第二十三条　职工应当参加职工基本医疗保险，由用人单位和职工按照国家规定共同缴纳基本医疗保险费。

　　无雇工的个体工商户、未在用人单位参加职工基本医疗保险的非全日制从业人员以及其他灵活就业人员可以参加职工基本医疗保险，由个人按照国家规定缴纳基本医疗保险费。

　　第二十四条　国家建立和完善新型农村合作医疗制度。

　　新型农村合作医疗的管理办法，由国务院规定。

　　第二十五条　国家建立和完善城镇居民基本医疗保险制度。

　　城镇居民基本医疗保险实行个人缴费和政府补贴相结合。

　　享受最低生活保障的人、丧失劳动能力的残疾人、低收入家庭六十周岁以上的老年人和未成年人等所需个人缴费部分，由政府给予补贴。

　　第二十六条　职工基本医疗保险、新型农村合作医疗和城镇居民基本医疗保险的待遇标准按照国家规定执行。

　　第二十七条　参加职工基本医疗保险的个人，达到法定退休年龄时累计缴费达到国家规定年限的，退休后不再缴纳基本医疗保险费，按照国家规定享受基本医疗保险待遇；未达到国家规定年限的，可以缴费至国家规定年限。

　　第二十八条　符合基本医疗保险药品目录、诊疗项目、医疗服务设施标准以及急诊、抢救的医疗费用，按照国家规定从基本医疗保险基金中支付。

　　第二十九条　参保人员医疗费用中应当由基本医疗保险基金支付的部分，由社会保险经办机构与医疗机构、药品经营单位直接结算。

　　社会保险行政部门和卫生行政部门应当建立异地就医医疗费用结算制度，方便参保人员享受基本医疗保险待遇。

　　第三十条　下列医疗费用不纳入基本医疗保险基金支付范围:

　　（一）应当从工伤保险基金中支付的；

　　（二）应当由第三人负担的；

　　（三）应当由公共卫生负担的；

　　（四）在境外就医的。

　　医疗费用依法应当由第三人负担，第三人不支付或者无法确定第三人的，由基本医疗保险基金先行支付。基本医疗保险基金先行支付后，有权向第三人追偿。

　　第三十一条　社会保险经办机构根据管理服务的需要，可以与医疗机构、药品经营单位签订服务协议，规范医疗服务行为。

　　医疗机构应当为参保人员提供合理、必要的医疗服务。

　　第三十二条　个人跨统筹地区就业的，其基本医疗保险关系随本人转移，缴费年限累计计算。

第四章　工　伤　保　险

　　第三十三条　职工应当参加工伤保险，由用人单位缴纳工伤保险费，职工不缴纳工伤保险费。

　　第三十四条　国家根据不同行业的工伤风险程度确定行业的差别费率，并根据使用工伤保险基金、工伤发生率等情况在每个行业内确定费率档次。行业差别费率和行业内费率档次由国务院社会保险行政部门制定，报国务院批准后公布施行。

　　社会保险经办机构根据用人单位使用工伤保险基金、工伤发生率和所属行业费率档次等情况，确定用人单位缴费费率。

　　第三十五条　用人单位应当按照本单位职工工资总额，根据社会保险经办机构确定的费率缴纳工伤保险费。

　　第三十六条　职工因工作原因受到事故伤害或者患职业病，且经工伤认定的，享受工伤保险待遇；其中，经劳动能力鉴定丧失劳动能力的，享受伤残待遇。

　　工伤认定和劳动能力鉴定应当简捷、方便。

　　第三十七条　职工因下列情形之一导致本人在工作中伤亡的，不认定为工伤：

　　（一）故意犯罪；

　　（二）醉酒或者吸毒；

　　（三）自残或者自杀；

　　（四）法律、行政法规规定的其他情形。

　　第三十八条　因工伤发生的下列费用，按照国家规定从工伤保险基金中支付：

　　（一）治疗工伤的医疗费用和康复费用；

　　（二）住院伙食补助费；

　　（三）到统筹地区以外就医的交通食宿费；

　　（四）安装配置伤残辅助器具所需费用；

　　（五）生活不能自理的，经劳动能力鉴定委员会确认的生活护理费；

　　（六）一次性伤残补助金和一至四级伤残职工按月领取的伤残津贴；

　　（七）终止或者解除劳动合同时，应当享受的一次性医疗补助金；

　　（八）因工死亡的，其遗属领取的丧葬补助金、供养亲属抚恤金和因工死亡补助金；

　　（九）劳动能力鉴定费。

　　第三十九条　因工伤发生的下列费用，按照国家规定由用人单位支付：

　　（一）治疗工伤期间的工资福利；

　　（二）五级、六级伤残职工按月领取的伤残津贴；

　　（三）终止或者解除劳动合同时，应当享受的一次性伤残就业补助金。

　　第四十条　工伤职工符合领取基本养老金条件的，停发伤残津贴，享受基本养老保险待遇。基本养老保险待遇低于伤残津贴的，从工伤保险基金中补足差额。

　　第四十一条　职工所在用人单位未依法缴纳工伤保险费，发生工伤事故的，由用人单位支付工伤保险待遇。用人单位不支付的，从工伤保险基金中先行支付。

　　从工伤保险基金中先行支付的工伤保险待遇应当由用人单位偿还。用人单位不偿还的，社会保险经办机构可以依照本法第六十三条的规定追偿。

　　第四十二条　由于第三人的原因造成工伤，第三人不支付工伤医疗费用或者无法确定第三人的，由工伤保险基金先行支付。工伤保险基金先行支付后，有权向第三人追偿。

　　第四十三条　工伤职工有下列情形之一的，停止享受工伤保险待遇：

　　（一）丧失享受待遇条件的；

　　（二）拒不接受劳动能力鉴定的；

　　（三）拒绝治疗的。

第五章　失　业　保　险

　　第四十四条　职工应当参加失业保险，由用人单位和职工按照国家规定共同缴纳失业保险费。

　　第四十五条　失业人员符合下列条件的，从失业保险基金中领取失业保险金：

　　（一）失业前用人单位和本人已经缴纳失业保险费满一年的；

　　（二）非因本人意愿中断就业的；

　　（三）已经进行失业登记，并有求职要求的。

　　第四十六条　失业人员失业前用人单位和本人累计缴费满一年不足五年的，领取失业保险金的期限最长为十二个月；累计缴费满五年不足十年的，领取失业保险金的期限最长为十八个月；累计缴费十年以上的，领取失业保险金的期限最长为二十四个月。重新就业后，再次失业的，缴费时间重新计算，领取失业保险金的期限与前次失业应当领取而尚未领取的失业保险金的期限合并计算，最长不超过二十四个月。

　　第四十七条　失业保险金的标准，由省、自治区、直辖市人民政府确定，不得低于城市居民最低生活保障标准。

　　第四十八条　失业人员在领取失业保险金期间，参加职工基本医疗保险，享受基本医疗保险待遇。

　　失业人员应当缴纳的基本医疗保险费从失业保险基金中支付，个人不缴纳基本医疗保险费。

　　第四十九条　失业人员在领取失业保险金期间死亡的，参照当地对在职职工死亡的规定，向其遗属发给一次性丧葬补助金和抚恤金。所需资金从失业保险基金中支付。

　　个人死亡同时符合领取基本养老保险丧葬补助金、工伤保险丧葬补助金和失业保险丧葬补助金条件的，其遗属只能选择领取其中的一项。

　　第五十条　用人单位应当及时为失业人员出具终止或者解除劳动关系的证明，并将失业人员的名单自终止或者解除劳动关系之日起十五日内告知社会保险经办机构。

　　失业人员应当持本单位为其出具的终止或者解除劳动关系的证明，及时到指定的公共就业服务机构办理失业登记。

　　失业人员凭失业登记证明和个人身份证明，到社会保险经办机构办理领取失业保险金的手续。失业保险金领取期限自办理失业登记之日起计算。

　　第五十一条　失业人员在领取失业保险金期间有下列情形之一的，停止领取失业保险金，并同时停止享受其他失业保险待遇：

　　（一）重新就业的；

　　（二）应征服兵役的；

　　（三）移居境外的；

　　（四）享受基本养老保险待遇的；

　　（五）无正当理由，拒不接受当地人民政府指定部门或者机构介绍的适当工作或者提供的培训的。

　　第五十二条　职工跨统筹地区就业的，其失业保险关系随本人转移，缴费年限累计计算。

第六章　生　育　保　险

　　第五十三条　职工应当参加生育保险，由用人单位按照国家规定缴纳生育保险费，职工不缴纳生育保险费。

　　第五十四条　用人单位已经缴纳生育保险费的，其职工享受生育保险待遇；职工未就业配偶按照国家规定享受生育医疗费用待遇。所需资金从生育保险基金中支付。

　　生育保险待遇包括生育医疗费用和生育津贴。

　　第五十五条　生育医疗费用包括下列各项：

　　（一）生育的医疗费用；

　　（二）计划生育的医疗费用；

　　（三）法律、法规规定的其他项目费用。

　　第五十六条　职工有下列情形之一的，可以按照国家规定享受生育津贴：

　　（一）女职工生育享受产假；

　　（二）享受计划生育手术休假；

　　（三）法律、法规规定的其他情形。

　　生育津贴按照职工所在用人单位上年度职工月平均工资计发。

第七章　社会保险费征缴

　　第五十七条　用人单位应当自成立之日起三十日内凭营业执照、登记证书或者单位印章，向当地社会保险经办机构申请办理社会保险登记。社会保险经办机构应当自收到申请之日起十五日内予以审核，发给社会保险登记证件。

　　用人单位的社会保险登记事项发生变更或者用人单位依法终止的，应当自变更或者终止之日起三十日内，到社会保险经办机构办理变更或者注销社会保险登记。

　　市场监督管理部门、民政部门和机构编制管理机关应当及时向社会保险经办机构通报用人单位的成立、终止情况，公安机关应当及时向社会保险经办机构通报个人的出生、死亡以及户口登记、迁移、注销等情况。

　　第五十八条　用人单位应当自用工之日起三十日内为其职工向社会保险经办机构申请办理社会保险登记。未办理社会保险登记的，由社会保险经办机构核定其应当缴纳的社会保险费。

　　自愿参加社会保险的无雇工的个体工商户、未在用人单位参加社会保险的非全日制从业人员以及其他灵活就业人员，应当向社会保险经办机构申请办理社会保险登记。

　　国家建立全国统一的个人社会保障号码。个人社会保障号码为公民身份号码。

　　第五十九条　县级以上人民政府加强社会保险费的征收工作。

　　社会保险费实行统一征收，实施步骤和具体办法由国务院规定。

　　第六十条　用人单位应当自行申报、按时足额缴纳社会保险费，非因不可抗力等法定事由不得缓缴、减免。职工应当缴纳的社会保险费由用人单位代扣代缴，用人单位应当按月将缴纳社会保险费的明细情况告知本人。

　　无雇工的个体工商户、未在用人单位参加社会保险的非全日制从业人员以及其他灵活就业人员，可以直接向社会保险费征收机构缴纳社会保险费。

　　第六十一条　社会保险费征收机构应当依法按时足额征收社会保险费，并将缴费情况定期告知用人单位和个人。

　　第六十二条　用人单位未按规定申报应当缴纳的社会保险费数额的，按照该单位上月缴费额的百分之一百一十确定应当缴纳数额；缴费单位补办申报手续后，由社会保险费征收机构按照规定结算。

　　第六十三条　用人单位未按时足额缴纳社会保险费的，由社会保险费征收机构责令其限期缴纳或者补足。

　　用人单位逾期仍未缴纳或者补足社会保险费的，社会保险费征收机构可以向银行和其他金融机构查询其存款账户；并可以申请县级以上有关行政部门作出划拨社会保险费的决定，书面通知其开户银行或者其他金融机构划拨社会保险费。用人单位账户余额少于应当缴纳的社会保险费的，社会保险费征收机构可以要求该用人单位提供担保，签订延期缴费协议。

　　用人单位未足额缴纳社会保险费且未提供担保的，社会保险费征收机构可以申请人民法院扣押、查封、拍卖其价值相当于应当缴纳社会保险费的财产，以拍卖所得抵缴社会保险费。

第八章　社会保险基金

　　第六十四条　社会保险基金包括基本养老保险基金、基本医疗保险基金、工伤保险基金、失业保险基金和生育保险基金。除基本医疗保险基金与生育保险基金合并建账及核算外，其他各项社会保险基金按照社会保险险种分别建账，分账核算。社会保险基金执行国家统一的会计制度。

　　社会保险基金专款专用，任何组织和个人不得侵占或者挪用。

　　基本养老保险基金逐步实行全国统筹,其他社会保险基金逐步实行省级统筹，具体时间、步骤由国务院规定。

　　第六十五条　社会保险基金通过预算实现收支平衡。

　　县级以上人民政府在社会保险基金出现支付不足时，给予补贴。

　　第六十六条　社会保险基金按照统筹层次设立预算。除基本医疗保险基金与生育保险基金预算合并编制外，其他社会保险基金预算按照社会保险项目分别编制。

　　第六十七条　社会保险基金预算、决算草案的编制、审核和批准，依照法律和国务院规定执行。

　　第六十八条　社会保险基金存入财政专户，具体管理办法由国务院规定。

　　第六十九条　社会保险基金在保证安全的前提下，按照国务院规定投资运营实现保值增值。

　　社会保险基金不得违规投资运营，不得用于平衡其他政府预算，不得用于兴建、改建办公场所和支付人员经费、运行费用、管理费用，或者违反法律、行政法规规定挪作其他用途。

　　第七十条　社会保险经办机构应当定期向社会公布参加社会保险情况以及社会保险基金的收入、支出、结余和收益情况。

　　第七十一条　国家设立全国社会保障基金，由中央财政预算拨款以及国务院批准的其他方式筹集的资金构成，用于社会保障支出的补充、调剂。全国社会保障基金由全国社会保障基金管理运营机构负责管理运营，在保证安全的前提下实现保值增值。

　　全国社会保障基金应当定期向社会公布收支、管理和投资运营的情况。国务院财政部门、社会保险行政部门、审计机关对全国社会保障基金的收支、管理和投资运营情况实施监督。

第九章　社会保险经办

　　第七十二条　统筹地区设立社会保险经办机构。社会保险经办机构根据工作需要，经所在地的社会保险行政部门和机构编制管理机关批准，可以在本统筹地区设立分支机构和服务网点。

　　社会保险经办机构的人员经费和经办社会保险发生的基本运行费用、管理费用，由同级财政按照国家规定予以保障。

　　第七十三条　社会保险经办机构应当建立健全业务、财务、安全和风险管理制度。

　　社会保险经办机构应当按时足额支付社会保险待遇。

　　第七十四条　社会保险经办机构通过业务经办、统计、调查获取社会保险工作所需的数据，有关单位和个人应当及时、如实提供。

　　社会保险经办机构应当及时为用人单位建立档案，完整、准确地记录参加社会保险的人员、缴费等社会保险数据，妥善保管登记、申报的原始凭证和支付结算的会计凭证。

　　社会保险经办机构应当及时、完整、准确地记录参加社会保险的个人缴费和用人单位为其缴费，以及享受社会保险待遇等个人权益记录，定期将个人权益记录单免费寄送本人。

　　用人单位和个人可以免费向社会保险经办机构查询、核对其缴费和享受社会保险待遇记录，要求社会保险经办机构提供社会保险咨询等相关服务。

　　第七十五条　全国社会保险信息系统按照国家统一规划，由县级以上人民政府按照分级负责的原则共同建设。

第十章　社会保险监督

　　第七十六条　各级人民代表大会常务委员会听取和审议本级人民政府对社会保险基金的收支、管理、投资运营以及监督检查情况的专项工作报告，组织对本法实施情况的执法检查等，依法行使监督职权。

　　第七十七条　县级以上人民政府社会保险行政部门应当加强对用人单位和个人遵守社会保险法律、法规情况的监督检查。

　　社会保险行政部门实施监督检查时，被检查的用人单位和个人应当如实提供与社会保险有关的资料，不得拒绝检查或者谎报、瞒报。

　　第七十八条　财政部门、审计机关按照各自职责，对社会保险基金的收支、管理和投资运营情况实施监督。

　　第七十九条　社会保险行政部门对社会保险基金的收支、管理和投资运营情况进行监督检查，发现存在问题的，应当提出整改建议，依法作出处理决定或者向有关行政部门提出处理建议。社会保险基金检查结果应当定期向社会公布。

　　社会保险行政部门对社会保险基金实施监督检查，有权采取下列措施：

　　（一）查阅、记录、复制与社会保险基金收支、管理和投资运营相关的资料，对可能被转移、隐匿或者灭失的资料予以封存；

　　（二）询问与调查事项有关的单位和个人，要求其对与调查事项有关的问题作出说明、提供有关证明材料；

　　（三）对隐匿、转移、侵占、挪用社会保险基金的行为予以制止并责令改正。

　　第八十条　统筹地区人民政府成立由用人单位代表、参保人员代表，以及工会代表、专家等组成的社会保险监督委员会，掌握、分析社会保险基金的收支、管理和投资运营情况，对社会保险工作提出咨询意见和建议，实施社会监督。

　　社会保险经办机构应当定期向社会保险监督委员会汇报社会保险基金的收支、管理和投资运营情况。社会保险监督委员会可以聘请会计师事务所对社会保险基金的收支、管理和投资运营情况进行年度审计和专项审计。审计结果应当向社会公开。

　　社会保险监督委员会发现社会保险基金收支、管理和投资运营中存在问题的，有权提出改正建议；对社会保险经办机构及其工作人员的违法行为，有权向有关部门提出依法处理建议。

　　第八十一条　社会保险行政部门和其他有关行政部门、社会保险经办机构、社会保险费征收机构及其工作人员，应当依法为用人单位和个人的信息保密，不得以任何形式泄露。

　　第八十二条　任何组织或者个人有权对违反社会保险法律、法规的行为进行举报、投诉。

　　社会保险行政部门、卫生行政部门、社会保险经办机构、社会保险费征收机构和财政部门、审计机关对属于本部门、本机构职责范围的举报、投诉，应当依法处理；对不属于本部门、本机构职责范围的，应当书面通知并移交有权处理的部门、机构处理。有权处理的部门、机构应当及时处理，不得推诿。

　　第八十三条　用人单位或者个人认为社会保险费征收机构的行为侵害自己合法权益的，可以依法申请行政复议或者提起行政诉讼。

　　用人单位或者个人对社会保险经办机构不依法办理社会保险登记、核定社会保险费、支付社会保险待遇、办理社会保险转移接续手续或者侵害其他社会保险权益的行为，可以依法申请行政复议或者提起行政诉讼。

　　个人与所在用人单位发生社会保险争议的，可以依法申请调解、仲裁，提起诉讼。用人单位侵害个人社会保险权益的，个人也可以要求社会保险行政部门或者社会保险费征收机构依法处理。

第十一章　法　律　责　任

　　第八十四条　用人单位不办理社会保险登记的，由社会保险行政部门责令限期改正；逾期不改正的，对用人单位处应缴社会保险费数额一倍以上三倍以下的罚款，对其直接负责的主管人员和其他直接责任人员处五百元以上三千元以下的罚款。

　　第八十五条　用人单位拒不出具终止或者解除劳动关系证明的，依照《[中华人民共和国劳动合同法](https://www.pkulaw.com/chl/7ab5e7d605f859e6bdfb.html#class=alink)》的规定处理。

　　第八十六条　用人单位未按时足额缴纳社会保险费的，由社会保险费征收机构责令限期缴纳或者补足，并自欠缴之日起，按日加收万分之五的滞纳金；逾期仍不缴纳的，由有关行政部门处欠缴数额一倍以上三倍以下的罚款。

　　第八十七条　社会保险经办机构以及医疗机构、药品经营单位等社会保险服务机构以欺诈、伪造证明材料或者其他手段骗取社会保险基金支出的，由社会保险行政部门责令退回骗取的社会保险金，处骗取金额二倍以上五倍以下的罚款；属于社会保险服务机构的，解除服务协议；直接负责的主管人员和其他直接责任人员有执业资格的，依法吊销其执业资格。

　　第八十八条　以欺诈、伪造证明材料或者其他手段骗取社会保险待遇的，由社会保险行政部门责令退回骗取的社会保险金，处骗取金额二倍以上五倍以下的罚款。

　　第八十九条　社会保险经办机构及其工作人员有下列行为之一的，由社会保险行政部门责令改正；给社会保险基金、用人单位或者个人造成损失的，依法承担赔偿责任；对直接负责的主管人员和其他直接责任人员依法给予处分：

　　（一）未履行社会保险法定职责的；

　　（二）未将社会保险基金存入财政专户的；

　　（三）克扣或者拒不按时支付社会保险待遇的；

　　（四）丢失或者篡改缴费记录、享受社会保险待遇记录等社会保险数据、个人权益记录的；

　　（五）有违反社会保险法律、法规的其他行为的。

　　第九十条　社会保险费征收机构擅自更改社会保险费缴费基数、费率，导致少收或者多收社会保险费的，由有关行政部门责令其追缴应当缴纳的社会保险费或者退还不应当缴纳的社会保险费；对直接负责的主管人员和其他直接责任人员依法给予处分。

　　第九十一条　违反本法规定，隐匿、转移、侵占、挪用社会保险基金或者违规投资运营的，由社会保险行政部门、财政部门、审计机关责令追回；有违法所得的，没收违法所得；对直接负责的主管人员和其他直接责任人员依法给予处分。

　　第九十二条　社会保险行政部门和其他有关行政部门、社会保险经办机构、社会保险费征收机构及其工作人员泄露用人单位和个人信息的，对直接负责的主管人员和其他直接责任人员依法给予处分；给用人单位或者个人造成损失的，应当承担赔偿责任。

　　第九十三条　国家工作人员在社会保险管理、监督工作中滥用职权、玩忽职守、徇私舞弊的，依法给予处分。

　　第九十四条　违反本法规定，构成犯罪的，依法追究刑事责任。

第十二章　附　　则

　　第九十五条　进城务工的农村居民依照本法规定参加社会保险。

　　第九十六条　征收农村集体所有的土地，应当足额安排被征地农民的社会保险费，按照国务院规定将被征地农民纳入相应的社会保险制度。

　　第九十七条　外国人在中国境内就业的，参照本法规定参加社会保险。

　　第九十八条　本法自2011年7月1日起施行。
